# Meningitis-Retention Syndrome as an Unrecognized Clinical Condition in Indian Scenario: Fall Through the Cracks

**DOI:** 10.7759/cureus.54910

**Published:** 2024-02-26

**Authors:** Gaurang K Gheewala, Deval U Surana, Anil Patel, Feral Daruwala

**Affiliations:** 1 Neurology, Gheewala Neuro Clinic, Surat, IND; 2 Medicine, Gheewala Neuro Clinic, Surat, IND; 3 Department of Respiratory Medicine, Smt. Rasilaben Sevantilal Shah Venus Hospital, Surat, IND; 4 Medical Writing, Gheewala Neuro Clinic, Surat, IND

**Keywords:** persistent leucocytosis, urinary retention, underactive detrusor, meningitis-retention syndrome, aseptic meningitis

## Abstract

Meningitis-retention syndrome (MRS) is a recently recognized condition marked by the concurrent occurrence of aseptic meningitis with acute urinary retention. We present the case of a 22-year-old man who presented with an undiagnosed fever with headache and urinary retention. Subsequent urodynamic testing revealed an underactive detrusor, resulting in insufficient contraction of the bladder during voiding despite normal sensation during bladder filling. Normal urinary voiding was successfully restored without the need for treatment within a 30-day timeframe. It is crucial to include MRS in the differential diagnosis of acute urinary retention. It is crucial to include MRS in the differential diagnosis of acute urinary retention. Despite the generally benign and self-remitting nature of MRS, the management of acute urinary retention is necessary.

## Introduction

Meningitis-retention syndrome (MRS) is an infrequent condition described by aseptic meningitis, often without a clearly identified causative agent and is linked to acute urinary retention [1]. The classic symptoms and neurological signs of aseptic meningitis may at times be mild or even absent, so the prominent manifestation frequently turns out to be isolated acute urinary retention [2]. Despite several documented cases in the literature, the actual prevalence of MRS remains underestimated. These factors obstruct an early diagnosis of MRS [3]. Herein, we present the case of a 22-year-old male patient who presented with a fever and headache. In the course of his hospitalization, he was diagnosed with aseptic meningitis and developed acute urinary retention. To the best of our knowledge, this is the first reported case of MRS from India.

## Case presentation

A 22-year-old male patient was admitted to Venus Hospital, Surat, with a one-month history of severe headaches. Upon physical examination, the patient’s blood pressure was 110/70 mmHg, heart rate was 109 beats per minute (bpm), and oxygen saturation was 97% on room air. His medical history was non-contributory, and he denied taking any medications. The patient received an intravenous paracetamol injection (150 mg) and experienced relief from the headache. Two days later, symptoms of urinary retention and constipation developed. The urodynamic tests showed an areflexic detrusor. During bladder filling, he felt a first sensation to void at 250 ml and a strong desire to void at 460 ml. However, there was a disappearance of sphincter electromyography (EMG) activity, and the detrusor contraction was not observable. The headache still persisted with increased intensity, and the patient was consulted by a neurophysician for further investigations.

At that time, routine blood examinations were normal, except for elevated levels of white blood cell counts (180 cells/mm^3^). Further, elevated levels of cell protein (105 mg/dl) and reduced levels of glucose (sugar) were found. The levels of cerebrospinal fluid (CSF) (Table 1), adenosine deaminase, and C-reactive protein (CRP) were within normal range.

**Table 1:** CSF analysis at different time intervals CSF, Cerebrospinal fluid; RBC, Red blood cells; WBC, White blood cells; Zn, Ziehl-Neelsen; ADA, Adenosine deaminase; AFB, Acid-fast bacillus; MTB, Mycobacterium tuberculosis.

Parameters	CSF-1	CSF-2	CSF-3	Reference range
Volume	4 ml	10 ml	5 ml	NA
Appearance	Colourless, clear	Slight turbid	Colourless, clear	Colourless, clear
Clot formation	Absent	Absent	Absent	NA
Protein (mg/dl)	105	159	110	0–50
Glucose (mg%)	31	30	101	40–70
RBC (µL)	Absent	Absent	Occasional	0–5
WBC (cells/mm^3^)	180	100	360	0-5
	Neutrophils 10% Lymphocytes 90%	Neutrophils 10% Lymphocytes 90%	Neutrophils 10% Lymphocytes 90%	Primarily lymphocytes
Gram stain	No pathogenic organism	No pathogenic organism	No pathogenic organism	NA
ZN stain	Negative for AFB	Negative for AFB	Negative for AFB	NA
ADA (unit/L)	<4	-	-	<40
X-Pert MTB	Negative	-	-	-

**Figure 1:**
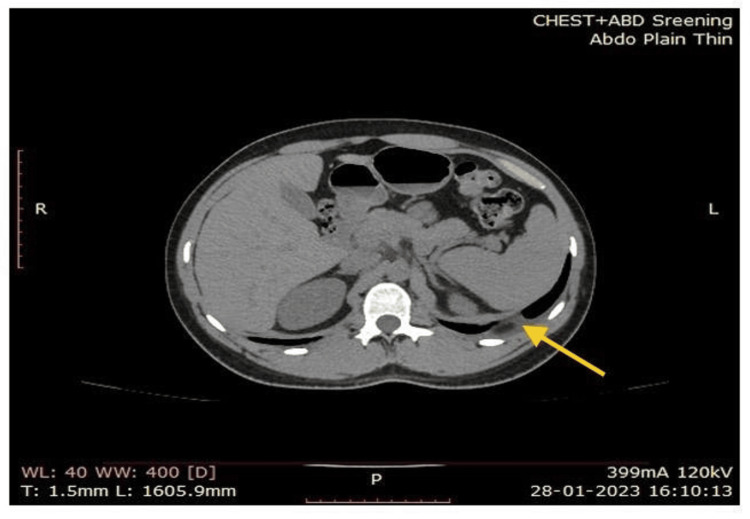
CT image showing minimal left pleural effusions

Computed tomography (CT) of the chest and abdomen revealed minimal left pleural effusions (Figure 1). Brain magnetic resonance imaging (MRI) did not reveal any features suggesting myelitis or radiculitis. Spine MRI shows no abnormalities. All CSF, blood, and urine cultures were tested negative for pyogenic bacteria, and CSF (Table 1) was negative in tuberculous culture. Brain magnetic resonance imaging (MRI) revealed no features suggesting myelitis or radiculitis.

Treatment was initiated with antibiotics, antacids, antiemetics, and other supportive medicines. The patient remained stable, experiencing only one fever spike and mild weakness. On the fourth day of hospitalization, the patient was shifted to the medical Intensive Care Unit (ICU) for altered sensorium and neck stiffness. The same management line, along with empirical AKT (rifampicin and isoniazid) and steroids, was continued. On the eighth day of hospitalization, the patient had a fever spike with stable vitals. The following day, he complained of difficulties in focusing on near vision. Brucella antibody, toxoplasma antibody, and tropical fever evaluation were negative. The antinuclear antibody (ANA) profile was negative. Repeat CSF examination showed persistent leucocytosis.

**Figure 2:**
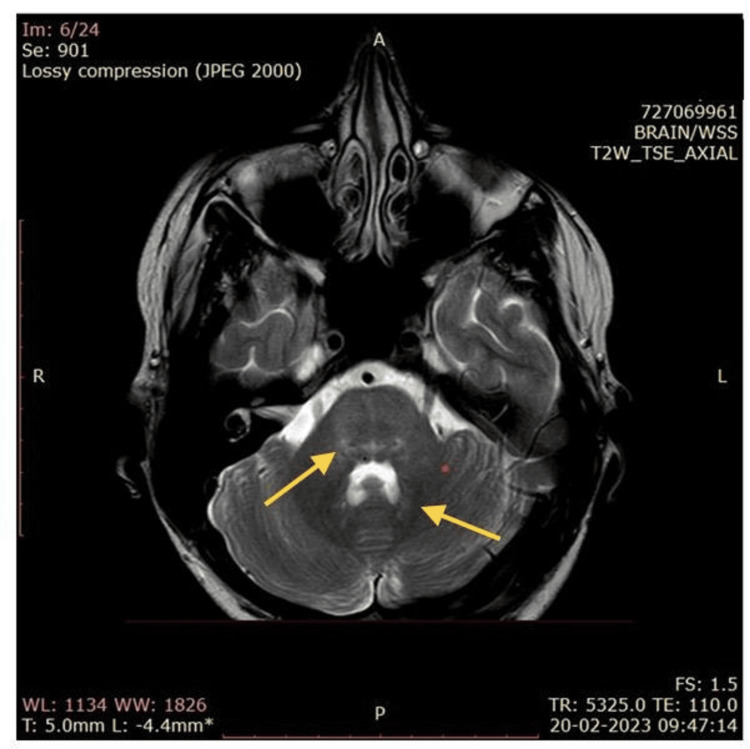
MRI of the head MRI showing minimal T2/flair hyperintense signal seen adjacent to the fourth ventricle at the dentate nucleus region of both cerebellum.

The meningitis panel (film array) was negative for bacteria, viruses, and cryptococcus. The autoimmune panel was negative for anti-NMDAR (N-methyl-D-aspartate (NMDA) receptor) antibody and voltage-gated potassium channel (VGKC) associated protein antibody (leucine-rich glioma-inactivated 1, LGI1), exclude the possibility of encephalitis and limbic encephalitis, respectively. The serum myelin oligodendrocyte glycoprotein (MOG) test was negative. Repeat MRI revealed minimal T2/flair hyperintense signal seen adjacent to the fourth ventricle at the dentate nucleus region of both cerebellum (Figure 2), indicative of inflammation, demyelination, or other pathological changes in the dentate nucleus region of the cerebellum. The patient was discharged and continued the same treatment, remaining in a stable condition.

However, after the second discharge, the patient was readmitted to the hospital with chief complaints of tremors in all four limbs, anxiety, and a history of fever spike at home. After reviewing multiple literature sources, we came to know that inflammatory neurologic diseases can also cause acute urinary retention. We identified this as a case of MRS, evidenced by leukocytosis, increased cell protein levels, decreased glucose levels in CSF examination, as well as the presence of detrusor hyperreflexia and the absence of viral titers. The treatment started with antibiotics, antacids, anti-emetics, intravenous immunoglobulin (IVIg), and other supportive medication. Tremors decreased, and the patient remained vitally stable with continued treatment.

## Discussion

Though aseptic meningitis is the most frequent condition, its co-occurrence with acute urinary retention is rare. Collectively, both of these conditions are known as MRS, a term first described by Sakakibara in 2005 [4]. Initially, patients presenting with symptoms such as fever and signs of meningeal irritation, including neck stiffness, altered sensorium, and headache, hinted at the possibility of pyrogenic meningitis and tuberculosis meningitis. However, the culture and gram stain for the targeted organisms were negative, leading to the exclusion of these conditions. In most reported MRS cases, the absence of encephalitic signs distinguishes it from acute disseminated encephalomyelitis (ADEM). Consistent with most of the published cases [4,5], CSF analysis in our case demonstrated persistent leukocytosis, reduced glucose content, and elevated protein levels indicative of aseptic meningitis. Subsequently, they developed urinary retention a week after the onset of neurological symptoms. Detrusor hyperreflexia was observed, and viral titers were absent. Given the concurrent urinary voiding difficulty and aseptic meningitis, a definite diagnosis of MRS is made. CSF adenosine deaminase (ADA) levels in non-infectious neurological diseases do not commonly increase, as seen in our case [6]. The absence of leg numbness and paresthesias also aids in distinguishing MRS from polyneuropathies, Guillain-Barre syndrome, and disorders affecting the lower motor neurons [4]. As CSF, blood, and urine cultures were negative in our patient, the exact cause of MRS remains undetermined.

Upon conducting urodynamic studies, it was observed that the patients exhibited an areflexic detrusor, leading to an impaired ability to properly contract the bladder during voiding [7]. Multiple theories have been suggested to elucidate detrusor hypofunction and urinary retention in MRS. Lesions affecting the central nervous system, either in the brain or the spinal cord, can result in detrusor areflexia, a usual phenomenon in conditions like transverse myelitis or ADEM [8]. We believe that the neurologic etiology of urinary retention in MRS is evident in our case, considering the absence of urologic diseases, including urinary tract infections. There is a compelling chronological association with urinary retention appearing either simultaneously or shortly after the onset of aseptic meningitis [2-4]. The precise location of the lesion responsible for urinary retention in MRS is yet to be determined. However, our hypothesis suggests that meningeal irritation may trigger an initial acute spinal shock, potentially affecting the innervation of the lower urinary tract (LUT).

Although a rare clinical syndrome, both neurologists and urologists may encounter MRS. It is considered a self-limited disease, and there is no definitive evidence to suggest that any treatment influences its clinical course. Although immune treatments such as steroids, antibiotics, and antiviral treatment have been attempted in most patients, their effectiveness remains unclear. In nearly all cases, the voiding function has recovered with an improvement of aseptic meningitis without the need for any specific treatment. The average reported time for the recovery of the voiding function is 10-32 days [9]. In our patients, a favorable prognosis was achieved within 30 days.

## Conclusions

We presented a case of MRS with subsequent urodynamic tests revealing an areflexic detrusor. Fortunately, the voiding function spontaneously recovered within 30 days without any specific treatment. Our case underscores the importance of recognizing MRS as a distinct neuro-urological condition. It is crucial to distinguish MRS from other conditions. Although MRS proves to be a benign and self-limiting condition, complete resolution needs time. Physicians should explain to patients with MRS that, as of now, no individuals have experienced permanent neurological damage to date, and all have successfully resumed their normal activities.
